# Supplementary material for: LARP4 mRNA codon-tRNA match contributes to LARP4 activity for ribosomal protein mRNA poly(A) tail length protection
Source: eLife. 2017 Sep 12;6:e28889. doi: 10.7554/eLife.28889 (PMC5626478; doi:10.7554/eLife.28889)
Supplement: Supplementary file 1. [file elife-28889-supp1.docx]

**Mattijssen et al.,**

**Supplementary Table File T1.**  Probes used for RNA detection

Hybridization buffer for antisense oligo’s is 6 x SSC, 2 x Denhardt’s, 0.5% SDS and 0.1 mg/ml yeast total RNA. * For certain tRNA probings, the probes were mixed in indicated ratios, since some genes differed slightly in sequence. Hybridization was carried out overnight.

| Target | Sequence 5’🡪3’ | Hybridization temp. |
| --- | --- | --- |
| LARP4 (excluding CRD region) | TTTAGATGCTACCTGCTC  CCTAAAATCATCCCATCAG  GCTTATGACTTGGTCTCAC  TCACTTCCTCTATTGGTG  CTTTGGGGCAGTTTTCAC  CCACCAGATGACCTAAAC  AAGGTAAGTTTGCTCCTG  CAAAACGAGTTCACCTGG  TAACTTTCGGGGTTCCTG  GAAGCCAGAATAATCCTTAC | 42 **°**C |
| Flag-Hind | AAGCTTGTCGTCATCGTCTTT | 47 **°**C |
| VA1 | GGTTCGACACGCGGGCGGTAACCG | 62 **°**C |
| GFP | CGTGCTGCTTCATGTGGTC  GTTCACCAGGGTGTCGCC  GGTCACGAACTCCAGCAGG | 50 **°**C |
| β-globin | CACCAGCCACCACCTTCTG  GGCAGCCTGCACCTGAGG  GCACCTTCTTGCCATGAGCC  CCTCACCACCAACTTCTTCCACATTC  GACAACCAGCAGCCTGCCCAG  CTTAGGATTGCTCATAACAGCATG | 50 **°**C |
| Rpl32 | GCTTCACAAGGGGTCTGAGG  AACTTCTTGGTTCTCTTTTTGACGATCTT  TTAATTTTGACATATCGGTCTGACTGGT  CCTCTGGGTTTCCGCCAGTTA  GTGAGCGATCTCGGCACAGTA | 52 **°**C |
| tRNA-Phe-GAA (D-loop)* | 87.5% CTCTCCCAACTGAGCTATTTCGGC  12.5% TCTCCCAACTGAGCTATCTCGGC | 55.3 °C |
| tRNA-Pro-AGG (D-loop)* | CATACCCCTAGACCAACGAGCC | 55.0 °C |
| tRNA-Thr-UGU (D-loop)* | 16.67% GCTTTAACCAACTAAGCCATAGAGCC  16.67% CTCTAACCACTGAGCTATGGAGCC  16.67% TCTAACCCCTGAGCTATAGGGCC  50% TCTAACCCCTGAGCTATGGAGCC | 54.4 °C |
| tRNA-Ser-GCU (D-loop) | TTAACCACTCGGCCACCTCGTC | 55.0 °C |
| tRNA-Ser-CGA (D-loop)* | 25% TTaACCACTCGGCCACCGTGAC  75% TTaACCACTCGGCCATCACAGC | 53.2 °C |
| tRNA-Tyr-GUA (T-loop)* | 50% CGAGCCGGAATCGAACCAGCG  8.33% CGAGCTGGAATCGAACCAGCG  8.33% CGAGCCGGAATTGAACCAGCG  33.33% CGAGCCGGATTCGAACCAGCG | 54.7 °C |
